# Supplementary material for: Lactoferrin Inhibits the Development of T2D-Induced Colon Tumors by Regulating the NT5DC3/PI3K/AKT/mTOR Signaling Pathway
Source: Foods. 2022 Dec 7;11(24):3956. doi: 10.3390/foods11243956 (PMC9777659; doi:10.3390/foods11243956)
Supplement: Supplementary file 1 [file foods-11-03956-s001.zip › Materials 2 NT5DC3 protein phosphorylation sites mutation data.pdf]

Wild1647 nt (**Normal control**)

ATGACCATGGCAGCGGCGGCGGTGGTGGCACGCGGGGCCGGGGCGAGGGCAGCGACAGCGGCGGCTTTG
CGGGGTGGCTGCGGGACCGCGGCTCGGGGGCGGCCGTGTGCGGGCCCCGCCCGGCCCTTGTGCACTGCA
CCCGGGACCGCCCCGGACATGAAGCGCTACCTGTGGGAGCGCTACCGGGAGGCGAAGAGAAGCACAGAA
GAATTGGTTCCTTCCATTATGAGCAACTTGTTGAATCCAGATGCCATTTTCTCAAACAATGAAATGAGC
CTGTCAGACATTGAAATCTATGGCTTCGATTATGATTACACCTTGGTGTTTTATTCAAAGCACCTCCAC
ACGCTGATATTTAATGCTGCACGGGACCTTCTCATCAATGAACACCGGTATCCAGCAGAAATCAGGAAG
TATGAGTATGACCCAAATTTTGCAATTCGTGGACTTCATTATGATGTACAGCGGGCAGTATTAATGAAG
ATCGATGCTTTTCATTATATCCAGCTGGGAACTGTCTACAGAGGCCTCAGTGTTGTCCCTGATGAAGAA
GTCATTGAAATGTACGAGGGGTCCCACGTGCCCTTGGAGCAGATGAGTGACTTTTACGGAAAGAGCTCT
CATGGAAACACGATGAAGCAGTTCATGGACATCTTCTCCCTGCCCGAGATGACCCTCCTGTCCTGCGTG
AATGAATACTTCCTCAAGAACAACATCGACTATGAGCCTGTGCATCTGTACAAAGATGTCAAGGATTCA
ATTCGAGACGTCCACATCAAAGGAATAATGTACAGAGCAATTGAAGCAGACATTGAAAAGTACATCTGC
TATGCTGAGCAGACCCGCGCAGTGTTGGCCAAACTGGCTGATCATGGCAAGAAGATGTTTCTCATCACC
AATAGCCCCAGTAGCTTTGTGGACAAAGGGATGAGTTATATCGTTGGGAAAGACTGGAGGGACCTGTTC
GATGTGGTCATTGTTCAGGCTGAGAAGCCAAACTTCTTTAATGATAAGCGGAGGCCTTTCCGAAAGATG
AATGAGAAAGGTGTCTTACTCTGGGATAAAATCCATAAGTTGCAGAAAGGCCAGATATACAAGCAGGGT
AATTTATATGAATTTTTGAAGCTTACTGGATGGAGAGGATCCAGAGTGTTGTATTTTGGTGACCATATA
TACAGTGACCTGGCGGATTTGACCCTAAAGCATGGCTGGAGGACTGGTGCAATCATCCCAGAGTTGAGA
TCTGAGCTCAAAATCATGAACACGGAGCAATACATTCAAACCATGACCTGGCTGCAGACCTTGACTGGC
TTATTGGAACAGATGCAGGTTCACAGAGATGCTGAGTCACAGCTGGTTTTGCAGGAGTGGAAAAAGGAA
AGGAAGGAGATGCGAGAAATGACCAAGAGTTTCTTCAATGCCCAGTTTGGAAGCCTGTTCCGCACAGAC
CAGAACCCAACCTACTTCCTAAGGCGCCTGTCGCGCTTCGCTGACATCTACATGGCGTCTCTGAGCTGC
CTCCTGAACTATGACGTCAGCCACACTTTCTACCCCCGGAGGACTCCACTGCAGCACGAACTGCCCGCC
TGGTCAGAAAGGCCCCCCACCTTCGGAACCCCTCTCCTGCAGGAGGCCCAGGCCAAGTAG

Thr6 mutant：1647 nt 6$_{th}$ Thr to proline（CCC）

ATGACCATGGCAGCGGCGGCGGTGGTGGCACGCGGGGCCGGGGCGAGGGCAGCGACAGCGGCGGCTTTG
CGGGGTGGCTGCGGGACCGCGGCTCGGGGGCGGCCGTGTGCGGGCCCCGCCCGGCCCTTGTGCACTGCA
CCCGGGACCGCCCCGGACATGAAGCGCTACCTGTGGGAGCGCTACCGGGAGGCGAAGAGAAGCCCCGAA
GAATTGGTTCCTTCCATTATGAGCAACTTGTTGAATCCAGATGCCATTTTCTCAAACAATGAAATGAGC
CTGTCAGACATTGAAATCTATGGCTTCGATTATGATTACACCTTGGTGTTTTATTCAAAGCACCTCCAC
ACGCTGATATTTAATGCTGCACGGGACCTTCTCATCAATGAACACCGGTATCCAGCAGAAATCAGGAAG
TATGAGTATGACCCAAATTTTGCAATTCGTGGACTTCATTATGATGTACAGCGGGCAGTATTAATGAAG
ATCGATGCTTTTCATTATATCCAGCTGGGAACTGTCTACAGAGGCCTCAGTGTTGTCCCTGATGAAGAA
GTCATTGAAATGTACGAGGGGTCCCACGTGCCCTTGGAGCAGATGAGTGACTTTTACGGAAAGAGCTCT
CATGGAAACACGATGAAGCAGTTCATGGACATCTTCTCCCTGCCCGAGATGACCCTCCTGTCCTGCGTG
AATGAATACTTCCTCAAGAACAACATCGACTATGAGCCTGTGCATCTGTACAAAGATGTCAAGGATTCA
ATTCGAGACGTCCACATCAAAGGAATAATGTACAGAGCAATTGAAGCAGACATTGAAAAGTACATCTGC
TATGCTGAGCAGACCCGCGCAGTGTTGGCCAAACTGGCTGATCATGGCAAGAAGATGTTTCTCATCACC
AATAGCCCCAGTAGCTTTGTGGACAAAGGGATGAGTTATATCGTTGGGAAAGACTGGAGGGACCTGTTC
GATGTGGTCATTGTTCAGGCTGAGAAGCCAAACTTCTTTAATGATAAGCGGAGGCCTTTCCGAAAGATG

AATGAGAAAGGTGTCTTACTCTGGGATAAAATCCATAAGTTGCAGAAAGGCCAGATATACAAGCAGGGT
AATTTATATGAATTTTTGAAGCTTACTGGATGGAGAGGATCCAGAGTGTTGTATTTTGGTGACCATATA
TACAGTGACCTGGCGGATTTGACCCTAAAGCATGGCTGGAGGACTGGTGCAATCATCCCAGAGTTGAGA
TCTGAGCTCAAAATCATGAACACGGAGCAATACATTCAAACCATGACCTGGCTGCAGACCTTGACTGGC
TTATTGGAACAGATGCAGGTTCACAGAGATGCTGAGTCACAGCTGGTTTTGCAGGAGTGGAAAAAGGAA
AGGAAGGAGATGCGAGAAATGACCAAGAGTTTCTTCAATGCCCAGTTTGGAAGCCTGTTCCGCACAGAC
CAGAACCCAACCTACTTCCTAAGGCGCCTGTCGCGCTTCGCTGACATCTACATGGCGTCTCTGAGCTGC
CTCCTGAACTATGACGTCAGCCACACTTTCTACCCCCGGAGGACTCCACTGCAGCACGAACTGCCCGCC
TGGTCAGAAAGGCCCCCCACCTTCGGAACCCCTCTCCTGCAGGAGGCCCAGGCCAAGTAG

Ser11 mutant：11th Ser to proline（CCC）

ATGACCATGGCAGCGGCGGCGGTGGTGGCACGCGGGGCCGGGGCGAGGGCAGCGACAGCGGCGGCTTTG
CGGGGTGGCTGCGGGACCGCGGCTCGGGGGCGGCCGTGTGCGGGCCCCGCCCGGCCCTTGTGCACTGCA
CCCGGGACCGCCCCGGACATGAAGCGCTACCTGTGGGAGCGCTACCGGGAGGCGAAGAGAAGCACAGAA
GAATTGGTTCCTTCCATTATGAGCAACTTGTTGAATCCAGATGCCATTTTCTCAAACAATGAAATGAGC
CTGTCAGACATTGAAATCTATGGCTTCGATTATGATTACACCTTGGTGTTTTATTCAAAGCACCTCCAC
ACGCTGATATTTAATGCTGCACGGGACCTTCTCATCAATGAACACCGGTATCCAGCAGAAATCAGGAAG
TATGAGTATGACCCAAATTTTGCAATTCGTGGACTTCATTATGATGTACAGCGGGCAGTATTAATGAAG
ATCGATGCTTTTCATTATATCCAGCTGGGAACTGTCTACAGAGGCCTCAGTGTTGTCCCTGATGAAGAA
GTCATTGAAATGTACGAGGGGTCCCACGTGCCCTTGGAGCAGATGAGTGACTTTTACGGAAAGCCCTCT
CATGGAAACACGATGAAGCAGTTCATGGACATCTTCTCCCTGCCCGAGATGACCCTCCTGTCCTGCGTG
AATGAATACTTCCTCAAGAACAACATCGACTATGAGCCTGTGCATCTGTACAAAGATGTCAAGGATTCA
ATTCGAGACGTCCACATCAAAGGAATAATGTACAGAGCAATTGAAGCAGACATTGAAAAGTACATCTGC
TATGCTGAGCAGACCCGCGCAGTGTTGGCCAAACTGGCTGATCATGGCAAGAAGATGTTTCTCATCACC
AATAGCCCCAGTAGCTTTGTGGACAAAGGGATGAGTTATATCGTTGGGAAAGACTGGAGGGACCTGTTC
GATGTGGTCATTGTTCAGGCTGAGAAGCCAAACTTCTTTAATGATAAGCGGAGGCCTTTCCGAAAGATG
AATGAGAAAGGTGTCTTACTCTGGGATAAAATCCATAAGTTGCAGAAAGGCCAGATATACAAGCAGGGT
AATTTATATGAATTTTTGAAGCTTACTGGATGGAGAGGATCCAGAGTGTTGTATTTTGGTGACCATATA
TACAGTGACCTGGCGGATTTGACCCTAAAGCATGGCTGGAGGACTGGTGCAATCATCCCAGAGTTGAGA
TCTGAGCTCAAAATCATGAACACGGAGCAATACATTCAAACCATGACCTGGCTGCAGACCTTGACTGGC
TTATTGGAACAGATGCAGGTTCACAGAGATGCTGAGTCACAGCTGGTTTTGCAGGAGTGGAAAAAGGAA
AGGAAGGAGATGCGAGAAATGACCAAGAGTTTCTTCAATGCCCAGTTTGGAAGCCTGTTCCGCACAGAC
CAGAACCCAACCTACTTCCTAAGGCGCCTGTCGCGCTTCGCTGACATCTACATGGCGTCTCTGAGCTGC
CTCCTGAACTATGACGTCAGCCACACTTTCTACCCCCGGAGGACTCCACTGCAGCACGAACTGCCCGCC
TGGTCAGAAAGGCCCCCCACCTTCGGAACCCCTCTCCTGCAGGAGGCCCAGGCCAAGTAG
